# Hierarchical Structure of the Program Used by Filamentous Fungi to Navigate in Confining Microenvironments

**DOI:** 10.3390/biomimetics10050287

**Published:** 2025-05-02

**Authors:** Gala Montiel-Rubies, Marie Held, Kristi L. Hanson, Dan V. Nicolau, Radu C. Mocanasu, Falco C. M. J. M. van Delft, Dan V. Nicolau

**Affiliations:** 1 Department of Bioengineering, McGill University, Montreal, QC H3A 0E9, Canada; gala.montielrubies@mail.mcgill.ca; 2 Department of Electrical Engineering & Electronics, University of Liverpool, Liverpool L69 3GJ, UK; m.held@liverpool.ac.uk (M.H.); mocanasu@yahoo.com (R.C.M.); 3 Industrial Research Institute, Swinburne University of Technology, Hawthorn, VIC 3122, Australia; kristi.hanson@senversa.com.au; 4 King’s College London, Faculty of Life Sciences & Medicine, School of Immunology & Microbial Sciences, Peter Gorer Department of Immunobiology, London SE1 1UL, UK; dan.nicolau@kcl.ac.uk; 5 MiPlaza, Philips Research Europe, 5656 AE Eindhoven, The Netherlands; falco.van.delft@molecularsense.com; 6 Molecular Sense Ltd., Liverpool L36 8HT, UK

**Keywords:** filamentous fungi, biological algorithms, space searching, space partitioning, microfluidics, optimization

## Abstract

The spatial navigation of filamentous fungi was compared for three species, namely *Pycnoporus cinnabarinus*, *Neurospora crassa* wild type and ro-1 mutant, and *Armillaria mellea*, in microfluidic structures. The analysis of the navigation of these filamentous fungi in open and especially confining environments suggests that they perform space exploration using a hierarchical, three-layered system of information processing. The output of the space navigation of a single hypha is the result of coordination and competition between three programs with their corresponding subroutines: (i) the sensing of narrow passages (remote- or contact-based); (ii) directional memory; and (iii) branching (collision-induced or stochastic). One information-processing level up, the spatial distribution of multiple, closely collocated hyphae is the result of a combination of (i) negative autotropism and (ii) cytoplasm reallocation between closely related branches (with anastomosis as an alternative subroutine to increase robustness). Finally, the mycelium is the result of the sum of quasi-autonomous sub-populations of hyphae performing distribution in space in parallel based on the different spatial conditions and constraints found locally. The efficiency of space exploration by filamentous fungi appears to be the result of the synergy of various biological algorithms integrated into a hierarchical architecture of information processing, balancing complexity with specialization.

## 1. Introduction

Fungal organisms can be found in every natural and artificial environment on Earth [1,2]. This ubiquity stems from the vastly diverse biological specializations of fungi, e.g., versatile and resilient decomposers, nutrient recyclers, pathogens, and symbionts, which, in turn, arises from their capacity to sense and appropriately respond to the surrounding environment [3]. The subset of filamentous fungi—which are themselves extremely diverse [4], and which often live in micro-confining environments, e.g., soil, wood litter and animal and plant tissues—evolved additional tools for spatial navigation for their survival, growth, and reproduction: space partitioning in open and quasi-open spaces and space searching in geometrically restricted environments.

The hypha, which is the prevailing unit of filamentous fungi [5], are long, cylindrical, multinucleated, branched threads that grow through unidirectional apical extension or tip growth [5,6,7]. The cell division of filamentous fungi results, eventually, in the formation of new growing points, forming a complex interconnected network of hyphae. This multilayer network, which after a certain complexity threshold constitutes the mycelium, is used by fungi to distribute its biomass to colonize space [8]. The sizes of the various constitutive modules of filamentous fungi, from individual hypha to full mycelia, vary extensively, ranging from nanometres for intracellular structures (less than 100 nm for the Spitzenkörper and other organelles [6]) to micrometres for hyphae (from a few µm in diameter to a few hundred µm for length [9,10]) and to centimetres and even many meters for mycelia [11]. This large range of sizes in which behavioural traits manifest requires different research paradigms and methodologies, making the development of an integrative perspective of fungal navigation difficult.

Without oversimplification, filamentous fungi must solve the optimization problem of how to maximize the rate and overall uptake of nutrients, which are distributed with an unknown spatial density and range, by covering the largest possible area at the optimum spatial density of the hyphae. This fundamental optimization problem, which is embedded in an interconnected network of optimization problems related to survival, growth, and reproduction, is affected by several factors. First, the rate of nutrient uptake modulates the production of cytoplasmatic biomass, which is essentially the ‘computing resource’ for solving the optimization problem, with a high rate of nutrient uptake accelerating the ‘computation’ process. Second, the spatial heterogeneity of nutrients translates, in the first instance, into a spatially uneven growth rate. Third, hyphae can extend over large, nutrient-depleted spaces, relying on the translocation of cytoplasmatic flow coming from other regions with a higher level of production of biomass [12,13,14]. Using the influential framework [15] of structuring the behaviour of living organisms like information processing units, we propose studying the program employed by filamentous fungi for the navigation of space, constituting their ‘software’, whereas the material structures and processes enabling this ‘software’ constitute their ‘hardware’.

In open or quasi-open spaces with no, remote, or very limited restrictions on growth, e.g., solid walls, the optimization problem is ‘solved’ by a sequence of *space partitioning* stages. The growth of each hypha divides the space into two mirrored regions, with subsequent branching resulting in the finer division of space. In confining environments, many of which harbor or are inhabited by filamentous fungi, the optimization problem has an additional set of restrictions, geometrical in nature, resulting in important behavioural differences, both quantitative and qualitative, compared with growth in open spaces. Consequently, space partitioning procedures are challenged, and filamentous fungi switch to more complex *space searching* procedures. From a quantitative point of view, fungal growth in micro-confining spaces is considerably slower, with more frequent branching [16,17]. From a qualitative point of view, it was shown that the growth of individual hyphae in confining environments is the result of a balance between two biological programs: collision-induced branching, with turgor pressure being the responsible biological hardware; and directional memory, with the responsible hardware being the complex formed by the Spitzenkörper and cytoskeletal microtubules [18].

The integrative study of the more complex biological software of filamentous fungi employed in space searching in confining environments should range from the observation of processes at the microscopic scale, such as a single hypha and several co-located hyphae, up to millimetre-range mycelia. Fortunately, microfluidics technology enabled the study of motility behaviour at the microscale, such as for bacteria [19,20], pollen tubes [21], plant roots [22,23], and nematodes [24]. More advanced studies used artificial *in vitro* micro-confining habitats, which mimic natural ones, to observe in a reproducible manner the behaviour and structure of filamentous fungi [25,26,27] and their interactions *in vivo* [9,10,18,28,29,30]. A shortcoming of these studies focusing on fungal growth in micro-confining environments is the limitation on the behaviour of individual hyphae, with only a few works [9,30] studying the inter-relationships between several hyphae in the process of space partitioning and searching.

The field of biomimetics, which started unceremoniously with the ‘reverse engineering’ of the Velcro tape, evolved dramatically in the last seven decades [31]. However, while biomimetic materials [32] and biomimetic chemistry [33] are almost ubiquitous, biomimetic information storage and processing have not reached its full potential [34]. Technology addresses problems through the manipulation of energy use, whereas biology uses information to ‘solve’ problems [35]. While “bio-inspired algorithms” are the subject of nearly five hundred articles (as per their title), the vastness of the size and diversity of biological information storage and processing suggests that harvesting of, rather than inspiration from, biological algorithms and procedures could lead to revolutionary developments if reverse-engineered and implemented.

The harvesting of biological algorithms used by filamentous fungi for space navigation in confining environments could follow several stages: (i) the qualitative description of their space searching programs, starting from individual hyphae to co-located hyphae up to millimetre-range mycelia, for several species with distinct behaviours, thus identifying common and different features; (ii) the quantitative description of biological software of interest, e.g., showing elements of computational efficiency; (iii) the transcription of biological programs into computer programs and comparison with similar artificial programs; and (iv) reverse engineering and implementing the biological programs that exhibit distinct efficiency. To this end, using our experimental results from three projects conducted over several years in multiple locations, but sharing the same experimental protocols and microfluidic structures, corroborated with observations from other reports, we contribute to the first stage of harvesting biological algorithms and procedures by proposing a broader, hierarchical perspective on the biological ‘software’ used by fungi for space navigation.

## 2. Materials and Methods

**Figure 1:**
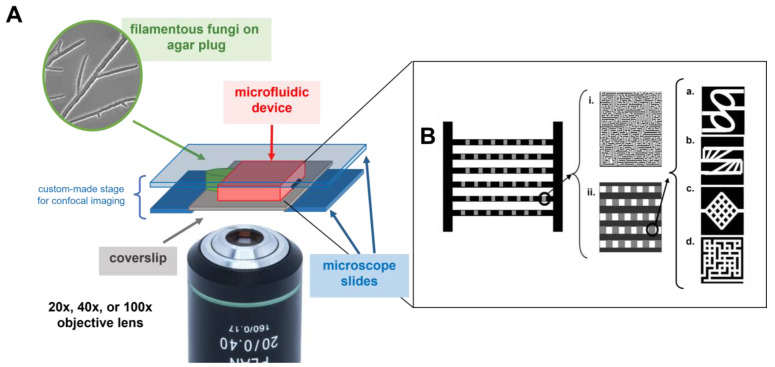
A schematic representation of the experimental system probing fungal space navigation in micro-confining environments. (**A**) The culture of the studied fungi was grown on malt agar, and a small plug was cut from the colony margin and placed at the entry of the confining geometry. The PDMS microfluidic device, which was bonded to a cover slip, was placed inside a custom-made stage, which made the visualization of fungal growth possible, even at high magnifications. (**B**) The hyphae entered through 2 mm wide ‘bus lanes’; each lateral channel, 1 mm wide, is a concatenation of open spaces and 1 × 1 mm test structures. These test structures were either large mazes (**i**), or more detailed structures comprising five parallel channels. The confining structures in (**ii**) are a concatenation of five 100 × 100 µm open spaces, alternating with 100 × 100 µm areas with various geometries made of 10 µm wide channels: loops (**a**), channels branching at different angles (**b**), symmetrical rectangular networks (**c**), and small mazes (**d**).

*Fungal species and culture. Neurospora crassa* wild type and mutant ro-1, *Pycnoporus cinnabarinus*, and *Armillaria mellea* were used as model organisms for the space navigation experiments in confining artificial microenvironments. Details regarding culturing fungi for experiments were reported elsewhere for *P. cinnabarinus* [36], *N. crassa* wild type and ro-1 mutant [17,37], and *A. mellea* [16]. In general, the fungal strains were maintained on 1.5% malt extract agar at 4 °C. Prior to each experiment, a fungal colony was sub-cultured by cutting a piece of the colony margin from an established colony and transferring it onto a fresh agar plate. This agar plate was then incubated at room temperature from 24 h up to 48 h. When the sub-cultured colonies had reached a minimum diameter of 3 cm, a small plug was cut and used for the assembly of experiments (experimental setup presented in Figure 1A).

*Fabrication of micro-confining environments*. An inverted confocal microscope (Figure 1A) was used to image fungal growth in a microfluidic chip (Figure 1B), which consisted of a network of parallel experimental geometries comprising 1 × 1 mm structures of 5 × 5 identical geometries separated by quasi-open spaces. These 100 × 100 µm chambers are connected by channels with 10 µm widths to fit individual hyphae presented with different geometries, i.e., loops, symmetrical rectangular networks, and small mazes. Additionally, a ‘big maze’ with an edge length of 1 mm, a constant channel depth of 10 μm, and channels of 20 μm widths was used to test the mycelium level of information processing.

The microfluidic devices were replicated from a positive-relief silicon master fabricated by standard photolithography techniques, followed by deep reactive ion etching (DRIE). Full details of the fabrication are provided in Supplementary Information SI1. The negative-relief replica was made following well-established procedures [38] using the base monomer of polydimethylsiloxane (PDMS) and a cross-linker (10:1 by weight) poured into the master and degassed for a few hours. The fully polymerized PDMS was then cured overnight at 65 °C to ensure full cross-linking of the polymer, and subsequently it was carefully peeled off. The patterned PDMS structure was then UV exposed and closed off by sealing it to a cover slip. Afterwards, the setup was immersed in nutrient-rich media (malt extract broth) and introduced to a desiccator to fill the channels. The sealed PDMS structure was set to a custom-made stage for confocal imaging. A fungal plug was placed in one of the lateral openings to allow for the introduction of hyphae into the test areas.

*Imaging.* Hyphal growth was imaged using two imaging systems. The initial experiments with *P. cinnabarinus* used an epifluorescence-inverted microscope, Olympus IX81, equipped with 20×, 40×, 60×, and 100× objectives (air, water, or oil) and equipped with a transmitted light differential interference contrast (DIC) attachment. The more advanced experiments used an Olympus Fluoview FV1000 Spectroscopic Confocal System (CSLM) mounted on the same Olympus IX81 microscope (Olympus Corporation, Tokyo, Japan). The system used an image-intensified CCD camera system (Coolview FDI, from Photonics Science Ltd., Robertsbridge, UK) controlled by Image-Pro Plus software (Ver. 5.0, Media Cybernetics, Rockville, MD, USA). The experiments with *N. crassa* wild type and ro-1 mutant and *A. mellea* used a confocal microscope Zeiss LSM 5 Exiter RGB (Carl Zeiss, Gottingen, Germany) with a built-in imaging system mounted on a Zeiss Axio Observer Z1 inverted microscope (Carl Zeiss, Gottingen, Germany), also equipped with objectives ranging from 10× to 100× (air, water, or oil) and with a DIC attachment.

## 3. Results and Discussion

### 3.1. Microfluidic Systems Testing the Space Navigation of Filamentous Fungi

*‘Reverse engineering’ of biological algorithms*. Bioinspired algorithms are a class of mathematical procedures that follow the structure of the procedures used by live biological entities [39,40,41] in ‘solving’ optimization problems related to the survival of individuals and of species. These algorithms, including those intended to solve optimal distribution in space [42,43], are indeed “inspired”, that is, they use general formulations of biological behaviours to construct computer programs with a similar structure. While this intellectual pathway leads to the rapid production of alternatives to *de novo*, human-developed algorithms, they also overlook the vast specificity and variety of the actual biological algorithms, which have evolved over billions of years in myriads of species. For instance, very recent work [44], which developed an optimizer inspired by fungal growth, correctly identifies generic biological ‘routines’, such as thigmotropism and nutrient- or toxin-driven chemotropism, but fails to account for well-documented [9,18,36], hypha-level ‘algorithms’, in particular collision-induced branching and directional memory. Another, less travelled, and arguably more arduous intellectual road is the ‘reverse engineering’ of biological algorithms, which would start progressing “bottom-up” from biology, instead of “top down” approach from mathematics and computer science. In the context of the ‘reverse engineering’ of the fungal programs for space searching, this work aims to contribute the first stage of the reverse engineering of a biological program.

*Microfluidics systems probing fungal space navigation.* Microfluidics technology has been, arguably, one of the most important achievements in biomedical engineering in the last two decades, with important accomplishments in lab-on-a-chip diagnostics [45] and high-throughput screening for drug discovery [46], but increasingly for the study of the behaviour of microorganisms at the microscale [25,27,47]. Microfluidics technology has, however, fundamental and inherent limitations. The most obvious limitation, largely caused by the necessity of visualising the phenomena hosted in microfluidic structures, is the obligated planarity of devices. Additionally, should visualization not be a necessity, the fabrication of natural 3D structures is not trivial, especially for a resolution below a few tens of microns. Also related to the need for imaging, which is essential for studies dealing with motility, microfluidics devices need to be transparent, which limits the materials than can be used for fabrication. While other transparent materials can be used, e.g., glass and silicon, PDMS-based technology is by far the most versatile, especially for cell growth studies given its biocompatibility, elasticity, and gas permeability characteristics [48].

*Designs of microfluidic structures.* As alluded above, the first stage of the process of the reverse engineering of biological algorithms requires the identification of the major software programs via observation *in vitro*, in this case at the microscopic level. Consequently, the various fungal species were exposed to microfluidic structures to test their basic responses to simple situations, rather than structures mimicking their actual microenvironment. These microfluidic devices enabled the study of fungal space navigation algorithms hierarchized from simple obstacles, e.g., vertical walls, to objects of increasing complexity, e.g., structures testing the directionality of growth in confining environments (Figure 1B(iia,b)), to geometries with increasing mathematical complexity, such as mazes with increased difficulty (Figure 1B(iic), iid, and i, respectively).

*Experimental conditions testing fungal space navigation.* One consequence of the necessity of first identifying the major fungal subroutines used for space searching in confined environments, i.e., stage (i) as described above, as opposed to their parametrisation, i.e., stage (ii), is the experimental treatment of environmental parameters. The parameters that are direct inputs to fungal growth, e.g., temperature, were kept constant and identical for all the species studied. Alternatively, if the environmental parameter is both affecting as well as being affected by fungal growth, i.e., nutrient concentration in confined spaces, we used malt extract broth, which contained nutrients in excess for fungal growth to avoid chemotaxis-driven behaviour and observe solely the impact of physical cues. The comprehensive exploration of the impact of the environmental conditions of fungal behaviour, while legitimate, is considerably beyond the scope of the present study. Consequently, where needed, when commenting on the ‘hardware’ responsible for fungal software’, we refer to the relevant comprehensive literature.

### 3.2. Selecting Fungal Species for the Study of Space Navigation

To describe the major space searching subroutines of the program used by filamentous fungi for space searching in confining environments, we performed a retrospective analysis of our previous works (Table 1). The selection of fungal species must address several key selection criteria. The selected species: (i) should be able to develop long hyphae, to allow for the long-term observation of space searching; (ii) should have similar dimensions, in particular hyphal diameters, to allow for comparative studies with identical test microfluidic structures; (iii) should colonize various microstructured environments, with the expectation that they will use variable space searching behaviours; (iv) should be amenable for growth in artificial media, following well-established experimental protocols; and finally (v) should not be pathogenic, especially to humans. These desideratus were fulfilled by the three fungal species, each with different ecological niches and characteristics. *A. mellea* is a pathogenic root rot fungus which infects woody ornamental trees, such as oak and pine, throughout the temperate and tropical regions of the world [49]. *P. cinnabarinus* is a common white rot fungus that degrades lignin from decaying wood through the production of extracellular phenoloxidases [50]. These are considered key species in forest ecosystems. *N. crassa* is a model filamentous fungus, which has been studied extensively due to its rapid growth and simple genetic manipulation. In nature, it can be found growing on burnt wood after forest fires [51]. Lastly, the hyphae of *N. crassa* ro gene mutants exhibit curled growth and abnormal nuclear distribution due to modifications in the microtubule-based motor cytoplasmic dynein or the associated dynactin complex [52]. *Aspergillus niger* also presented interesting space searching patterns (Figure SI1). However, while this species is considered safe for food production [53], its further study ceased because of the remotely possible production of toxins [54].

### 3.3. Space Navigation at the Individual Hypha Level

#### 3.3.1. Sensing of Narrow Entries

**Figure 2:**
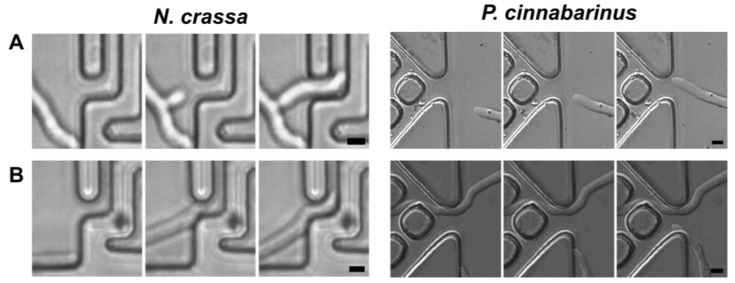
Remote and contact-based sensing of narrow passages by *N. crassa* and *P. cinnabarinus*. Exemplifying timelapses for both fungal species using remote sensing (**A**) or contact sensing (**B**). Scale bars: 5 μm.

When the hyphae approached a confining structure from a more open space, that is, transitioning from space partitioning to space searching, two behaviours were observed: contactless ‘remote sensing’, and wall contact-based searching for a narrow passage. The hyphal exploration of narrow passage subroutines is presented in Figure 2 for both *N. crassa* and *P. cinnabarinus.*

*Remote sensing*

Software. Remote sensing occurred when a narrow passage was located at a longer distance from the apex compared with the hyphal diameter and when the leading hypha re-aligned its growth directionality towards the entry (Supplementary Movie SM 01). The remote sensing behaviour was observed more often for both species if the entry to the narrow passage was located closer to the tip of the leading hypha. In these cases, remote sensing was often associated with the emergence of a ‘daughter’ hypha, with its initial direction pointing towards the narrow passage point (Supplementary Movie SM 02).

Hardware. While the fungal mechanisms for remote sensing are yet to be fully described, and thus understood, several known elements can help us to hypothesize an enabling hardware system. First, hyphal walls possess chemical and pressure sensors essential for chemotaxis, e.g., for oxygen, waste products, quorum sensing molecules [3,55,56], and pressure taxis [57,58], respectively. Second, hyphae generate a longitudinal pH gradient, with the medium surrounding the apex being more acidic than the bulk phase and the medium adjacent to the more mature hyphae being more alkaline [59,60]. This ‘pH cloud’, which would have an isotropic, spherical shape surrounding the hyphal tip when growing in open spaces, will be deformed by the confining space, resulting in an anisotropic chemical landscape. Third, the ‘piston-like’ movement of the hypha in closed environments can create a pressure gradient, with less pressure towards the narrow open entry. Whatever its mechanism, this self-generated chemical or pressure anisotropy could be mapped by fungal sensing, similarly with ‘geolocation’ used by bats. Additionally, *P. cinnabarinus* presented more instances of remote sensing than *N. crassa*. We hypothesize this could also be explained by the difference in their cell walls, as *P. cinnabarinus* could have a mechanically stronger cellular wall needed for the penetration of wood litter [61], translating into longer ‘free-standing’ hyphae. Additionally, the *N. crassa* ro-1 mutant, which had a defective cytoskeleton system, and thus is mechanically weak internally, did not present any discernible remote sensing and exhibited only close contact with the walls, ‘flowing’ toward any available opening.

*Contact-based sensing*

Software. This subroutine operates when the tip of an individual hypha encountered a wall and slid along until a narrow passage was found. This mechanism has been described as thigmotropism [62]. *P. cinnabarinus* followed the wall only with the hyphal tip, with the rest of the hyphae staying curved and at some distance from the wall (Supplementary Movies SM 03 and 04). This ‘finger-like’ contact sensing resulted in some operational difficulties when encountering a passage, e.g., hypha ‘snapping’ or laterally branching in the narrow entry. Conversely, *N. crassa* conformally followed the wall profile, ‘flowing’ seamlessly in narrow passages. For *A. mellea,* it appeared that this species uses almost exclusively contact sensing, even for long trajectories (Supplementary Movie SM 05).

Hardware. Physical cues, such as the topography of a surface, can initialize different cell types and structures in pathogenic fungi [63]. Several studies of thigmotropism in different fungal species [62,64,65,66] demonstrated their ability to sense micrometre-sized structures on the hyphal tip, followed by the redirection of hyphae. The hardware hypothesized to be used in this subroutine could be attributed to biochemical cascades produced by the deformation of the cell wall membrane and the change in the force balance between motor-dependent forces and turgor-generated outward forces [58].

#### 3.3.2. Directional Memory

As comprehensively reported before, the space searching of individual hyphae in confined spaces for *P. cinnabarinus* [36], *N. crassa* [17,18,37], and *A. mellea* [16] present the characteristic features of ‘directional memory’, that is, returning to the initial direction of growth immediately or soon after the disappearance of the object blocking growth.

**Figure 3:**
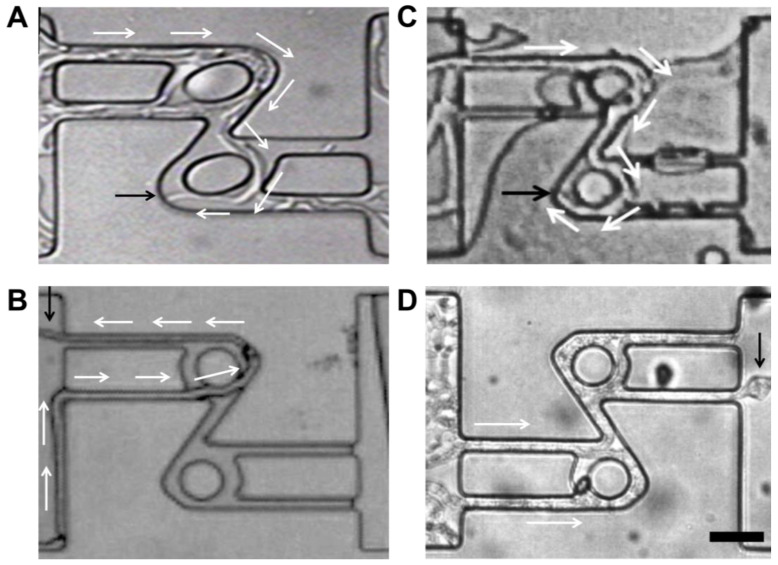
Mechanical resilience of directional memory when challenged by nestling in circular geometry. Fungal growth occurred from left to right; white arrows indicate growth direction, while black arrows indicate the final position of tracked hyphae. (**A**) Circular loop causes *P. cinnabarinus* to branch inside channels. The leading hypha becomes redirected by the circular pillar. (**B**) Loop geometries gradually deflect the directionality of the leading hypha of *A. mellea* without causing any side-branching and rerouting to opposite initial direction of growth. (**C**) *N. crassa* presents similar behaviour to *P. cinnabarinus* in the loop structure. (**D**) Hyphae of *N. crassa* ro-1 lack directional memory routine, and branches abundantly occupying all available space. Scale bar: 20 μm.

Software. Directional memory presented species-specific characteristics. For example, *P. cinnabarinus* presented long-lasting directional memory, which enabled it to find the maze exits more quickly and with a high rate of success (Supplementary Movie SM 02). Additionally, the directional memory of *P. cinnabarinus* appeared to be resilient, although it was eventually challenged by convolutedly bent structures (Figure 3A). *N. crassa* also presented an exceptionally long directional memory, lengthwise (and consequently timewise), lasting for paths many tens of times longer than the hyphal diameter. However, *N. crassa* appeared to possess a less resilient directional memory, as its hyphae made U-turns in the microfluidic channels, challenging its directional memory if away from the initial point of growth (Figure 3C). The *N. crassa* ro-1 mutant did not exhibit directional memory in any of the microfluidic networks, as these hyphae can turn back toward their initial growth point, even after encountering structures that do not oppose directional memory (Figure 3D). Finally, *A. mellea* has a short span directional memory as it became lost after a few micrometres when encountering the roundabout (Figure 3B).

Hardware. From a biological hardware perspective, it was shown for *N. crassa* [18] that directional memory is the result of the operation of a synergetic system comprising the Spitzenkörper, which operates as a biological gyroscope preserving initial branch directionality, and the microtubule cytoskeleton, which consolidates this memory.

#### 3.3.3. Branching

Given that filamentous fungi are sessile organisms, branching appears to be the only alternative to cell division for efficiently colonizing space using two subroutines for both space partitioning and space searching [7].

*Collision-induced branching*

Software. As reported before, in confined spaces, the hyphae of both *P. cinnabarinus* [36] and *N. crassa* [17,18] performing space partitioning switched to space searching of the available space, with branching triggered by contact with the solid obstacles opposing fungal growth. Even if branching increases the number of alternative search directions, excessive branching can lead to sub-optimal space searching in confining spaces [16]. When exposed to micro-confining environments, both *P. cinnabarinus* and *N. crassa* dramatically increased their branching frequency compared with that in open environments, as also observed previously for other species [9,67]. Consequently, the allocation of the cytoplasm within more frequent branches inherently resulted in an equivalent decrease in apical growth.

**Figure 4:**
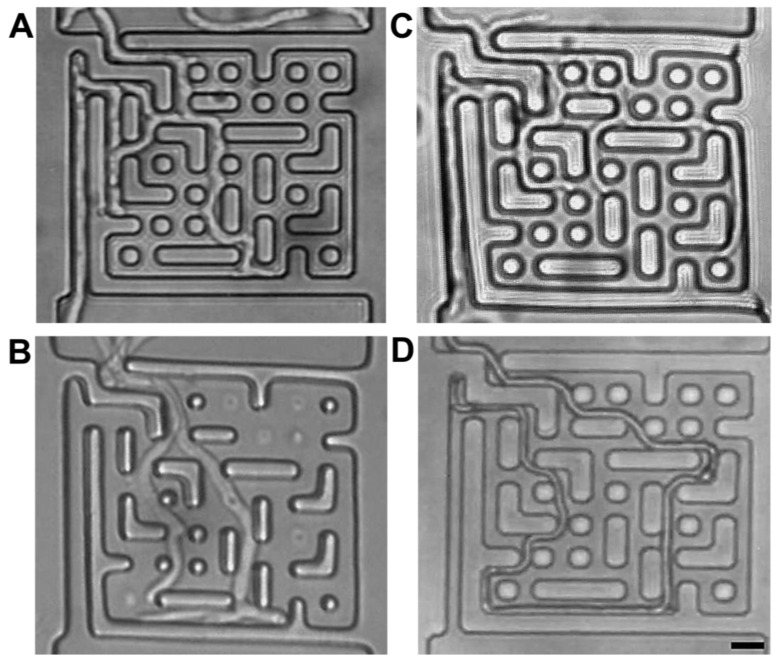
Branching patterns of different fungal species in microfluidic maze. (**A**) *N. crassa*, (**B**) *P. cinnabarinus* (**C**) *N. crassa* ro-1 mutant, and (**D**) *A. mellea*. Scale bar: 10 μm.

When colonizing confined microstructures, the branching patterns differ among species. *P. cinnabarinus* [36] almost never branched at the tip of the leading hypha, but behind at lengths equivalent to several hyphal diameters (Figure 4B, and Supplementary Movie SM 06). *N. crassa* branched both by apical splitting and by collision-induced branching, as presented in Figure 4A and Supplementary Movies SM 07. The *N. crassa* ro-1 mutant branched in a disorganized manner, increasing its hyphal density and nesting towards the wall that it was following (Figure 4C). Lastly, regarding the diversity of the particularities of collision-induced branching, *A. mellea* [16] very rarely branched, even in very complex spaces, i.e., dead-ends and mazes (Figure 4D and Supplementary Movies SM 05, SM 08 and SM 10). It also appears that collision-induced branching exhibits elements of ‘memory’, as evidenced by the gradual decrease in the branching frequency once the hyphae were out of the confining spaces into more open spaces (Figure SI2). This species-specific variability regarding branching in confining spaces was also described in a recent microfluidics-based study [67], which compared 14 fungal strains occupying various ecological niches, e.g., saprotrophs and pathogens (in animals, including humans, and plants). It was found that within the species studied, there were two distinct types of confinement-induced branching: high-frequency branching, in which all possible paths were explored, like for both *P. cinnabarinus* and *N. crassa*; and low-frequency branching, in which only one or two paths were explored, similar to *A. mellea*.

Hardware. As argued before [7,18], the hardware for collision-induced branching appears to be driven by turgor pressure. However, this could also be modulated by the mechanical strength of the hyphal membrane, as evidenced by the difference in the locations of branching of the leading hypha. More in-depth experiments quantifying cell wall thickness and strength, as well as species-specific turgor pressure measurements, could help the quantification and understanding of this relationship.

*Stochastic branching*

Software. Branching in fungi serves two main purposes: increasing the surface area of the colony to thereby increase nutrient assimilation, as well as mediating the different mechanisms of the exchange of nutrients and signals within the mycelium [68]. In highly nutritive environments with smooth surfaces, such as malt agar plates, this subroutine results in fractal geometries [69,70,71,72,73].

Hardware. Branching, triggered by internal mechanisms, is explained as a combination of intracellular signals, circadian clock-associated proteins, calcium ions, actin cytoskeleton presence, stochasticity, and the accumulation of internal pressure [7,74], all of which would still remain “on” in confining spaces. The characteristic polarization of growth due to tip extension or apical dominance protects fungal colonization from chaotic growth.

### 3.4. Space Navigation at the Co-Located Hyphae Level

The various space searching-related subroutines performed by individual hyphae, as described above, result in the modification of the microenvironment they are growing in, i.e., restricting the availability of space, altering the chemical landscape, and possibly altering the local pressure. These changes further affect the behaviour of the neighbouring hyphae if confined in the same space. Despite this general framework, the behaviours of few co-located hyphae growing in the same closed environment are markedly different than those of individual branches, determined by the nature of their intercommunication, which can be *extracellular*, taking the form of negative autotropism, or *intracellular*, taking the form of cytoplasm reallocation.

#### 3.4.1. Negative Autotropism

When confined in mesoscale confining geometries, that is, with lateral dimensions allowing for the moderate unrestrained growth of at least two hyphae at the same time, the hyphae often diverted their growth from their initial direction away from each other. Sometimes, even this behaviour contradicted the expected outcome dictated by directional memory. This behaviour, termed negative autotropism [75], is commonly observed in open, flat environments, as a self-avoidance reaction amongst neighbouring hyphae, resulting in the ramification of evenly spaced cells to maximize the occupation of a substrate [76]. Depending on space availability and the initial direction of growth of the interacting hyphae, two extreme variants of negative autotropism were observed.

*Side-by-side negative autotropism*

**Figure 5:**
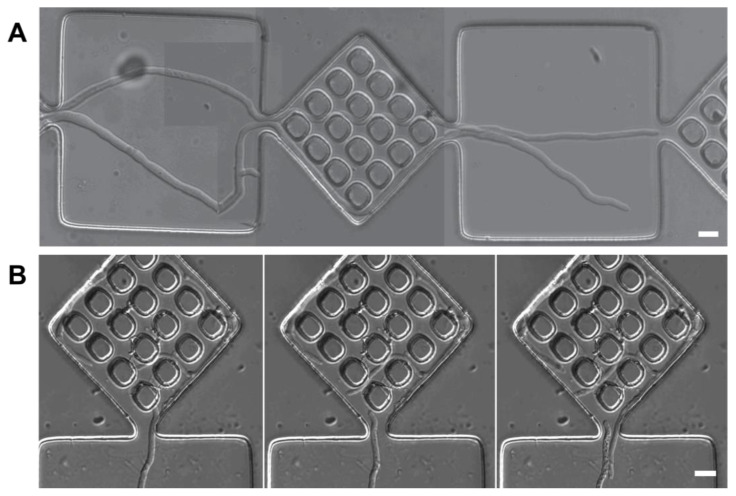
Negative autotropism in confining structures of *P. cinnabarinus*. (**A**) Stitched image of *P. cinnabarinus* in a 100 × 100 μm chamber advancing through confined structure and moving into open space again. (**B**) Extreme example of negative autotropism. When advancing hypha encounters several hyphae from opposite side, it ceases its apical extension and retracts its cytoplasm. Scale bars: 10 μm.

Software. In side-by-side negative autotropism, two (or more) hyphae grow in the same general direction, and they often tend to redirect their growth laterally away from each other if the structures allow this divergence (Supplementary Movies SM 06 and SM 09 and Figure 5 for *P. cinnabarinus*, and Supplementary Movie SM 10 for *A. mellea*). In many instances, negative autotropism, which contradicted the directional memory of the adjacent hyphae, led to blockages in the corners, followed by obligated branching, buckling, and even cessation of growth. Importantly, other than the space needed for this behaviour to manifest, the diverging hyphae must be unrelated even if growing side-by-side, that is, their common parent needs to be located at a longer distance behind. *A. mellea* presented a particular negative autotropism behaviour, which manifested after the hyphae exited from very narrow passages (although this could be a simple manifestation of the directional memory of individual hyphae), but not in larger spaces (Supplementary Movie SM 10).

*Head-on negative autotropism*

Software. An extreme case was observed for *P. cinnabarinus* when two hyphae ‘met each other’ coming from opposite directions. In this instance, one of the hyphae started to retreat to the extent possible by its already mature cellular walls and started transporting its cytoplasm backwards (Figure 5B and Supplementary Movie SM 11). Interestingly, the hyphae of *N. crassa* did not present prevalent self-avoiding traits, and consequently head-on negative autotropism was extremely rare.

Hardware. Negative autotropism for filamentous fungi was studied for growth in open spaces based on the excretion of local inhibitory cues, as well as searching for available oxygen. These studies show that fungi prevent the appearance of further protrusions when there is a newly formed branch, as well as avoiding growth in areas with oxygen-depleted zones [7,76].

#### 3.4.2. Cytoplasm Reallocation

While fungal growth requires the transport of biomass towards the hyphal apex in the direction of growth, cytoplasm reallocation consists in the biomass flowing backwards compared with the initial direction of growth. This behaviour was observed for both *P. cinnabarinus* (Supplementary Movies SM 03, SM 06 and SM 11) and to a lesser extent for *N. crassa*. Because its growth is essentially free of collision-induced branching, cytoplasm reallocation was not observed for *A. mellea*.

**Figure 6:**
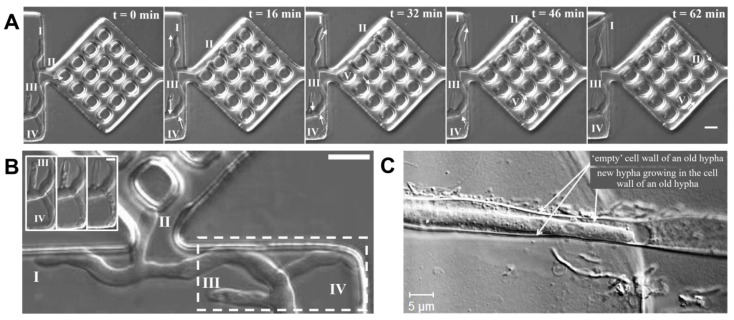
Cytoplasm reallocation between closely related branches for *P. cinnabarinus* and *N. crassa*. (**A**) *P. cinnabarinus* hyphae reallocating cytoplasm contents when exploring symmetrical rectangular network. As hypha II, which branches to secondary hypha (V) advance, hyphae III and IV disappear to maintain growth in the searching front. (**B**) Zoomed-in image of *P. cinnabarinus* cytoplasm reallocation process, demonstrating the translocation of computational resources in approximately one hour. Scale bars: 10 μm. (**C**) Extreme case of cytoplasm reallocation creates opportunity for young hypha of *N. crassa* to grow in an old empty cell wall of a hypha that was evacuated by retraction.

Software. Cytoplasm reallocation often occurred when all the other space searching subroutines failed to produce viable alternatives for hyphal growth. For instance, after several newborn branches of *P. cinnabarinus* failed to progress through a nearby narrow passage, one ‘winning’ hypha found a viable alternative to growth, and then the ’sibling’ hyphae ‘dried up’, supporting the new direction of growth (Figure 6A,B and Supplementary Movie SM 06). In an extreme case, albeit in an open environment, the backwards transport of cytoplasm was so complete it allowed for the growth of another hypha inside the empty tubular structure of the empty one (Figure 6C). While normal cytoplasm transport towards the general direction of growth is the basis for *long-distance* resource distribution, colonization, and biomass recycling, cytoplasm reallocation is operating as a last attempt to run space searching *locally*, even after the back-up collision-induced branching fails at providing exploratory avenues.

Hardware. From a biological hardware perspective, hyphae permit the translocation of resources in a heterogenous environment, enabling the circulation of carbohydrates, amino acids, and phosphate throughout the mycelium [77]. When monitoring the growth of mycorrhizal fungi, there was an intense signal from cytoskeletal proteins in the mycelium when there was the retraction of cytoplasm [78]. However, osmotic pressure gradients drive bulk cytoplasmic streaming, implying that cytoskeletal proteins and microtubules might have a secondary role. Hyphal differentiation (especially hyphal architecture) due to colony maturation and tissue organization has been hypothesized to allow for differences in retrograde translocation and the reallocation of nutrients from sources to sinks in different fungal species. The cascading signals responsible for the major uptake of water from the environment, or an increasing secretion or production of molecules to modify the osmotic environment inside the hyphae, as well as the signals governing fusing events in the mycelium are still obscure. More research needs to be conducted to determine the different signaling processes that are involved and the different characteristics responsible for this strategy of space searching.

*Anastomosis*

Software. In a sense, anastomosis, where a single hypha fuses with a collocated one, is an essential step for increasing the functional robustness of the network. When predators and fungivores eat or damage the mycelium, forming redundant loops [79], this allows for the rapid reallocation of resources in response to local demands [8]. Additionally, this process is important for intrahyphal communication, the translocation of water and nutrients, as well as genetic material, and overall general homeostasis within an individual colony [80].

Hardware. When two hyphae fuse with each other through cell wall breakdown and immediate membrane merging, cytoplasmic continuity is created to reinforce the robustness of the system [81]. This process is dependent on the maturation and density of the colony, with more fusion events in the colony interior, or older hyphae, than the periphery. Hyphae presented increased positive autotropism, with a distinct branching pattern that fused through tip-to-tip or tip-to-side mechanisms [80]. Fungal fusion has been proved through the use of different mutants to be possible due to the regulation of complex signaling networks, such as the mitogen-activated protein kinase (MAPK), cell wall integrity (CWI), and pheromone response pathways [82].

### 3.5. Space Navigation at the Mycelium Level

To test overall mycelium behaviour, a 10 × 10 larger maze was used. This layout locally confined the hyphae two times less, with 20 μm width channels, but had a vastly increased channel density, around 22 mm of the total available path length for exploration. The complexity of the structure, in addition to the presence of dead ends and higher obstacle heterogeneity, consists of a single solution path of 4.5 mm in length from the entry found on the top right and the exit found on the bottom left of the structure. This maze would then increase he space searching challenge for the fungi at least 10 times compared to the smaller maze and the different channel configuration structures.

**Figure 7:**
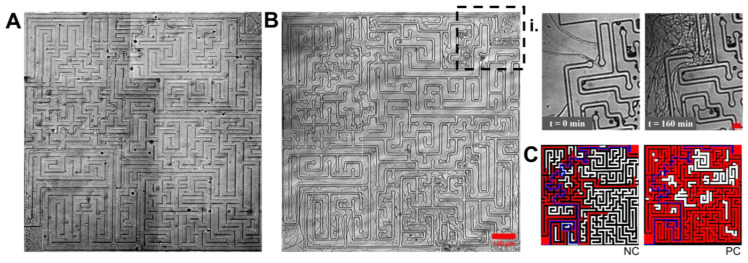
Space navigation at the mycelium level for *N. crassa* and *P. cinnabarinus*. (**A**) *N. crassa* in a 1 × 1 mm maze. (**B**) *P. cinnabarinus* exploring the same structure. (**i**) Zoomed-in images of the inlet, highlighted as a dotted rectangle for *P. cinnabarinus*. This shows the evolution of the hyphae clogging a dead end, and then finding an alternative route to continue colonizing the space in around 2 h. Scale bar: 20 um. (**C**) Binarized maze and fungal growth (red) for both *N. crassa* (NC) and *P. cinnabarinus* (PC). The only solution path in the maze is presented in blue.

Software.  *N. crassa* and *P. cinnabarinus* successfully ‘solved’ this larger structure (Figure 7A,B, respectively). It appears that this success was more the result of small-scale, ‘localized’ solutions of parts of the maze, without any apparent ‘global’ coordination by a meta-program for space searching at the mycelium level, as suggested by the clustering of hyphal biomass for both *N. crassa* and *P. cinnabarinus* (Figure 7C). It is important to note that the complexity and type of mycelium is species-specific, with possible consequences for the mechanisms of translocation within their respective networks. Some species are characterized by having opportunistic, far-reaching explorative hyphae, while others colonize new territories by short-range foraging, growing dense mats of hyphae, and advancing slowly as a united front [9]. In our experimental setup, *N. crassa* growth is more compact, while *P. cinnabarinus* presents a more resource intensive search.

Hyphal morphology changes with age and the developmental state of the mycelium. As time progresses, more mature networks experience the significant loss of connections, the thinning of the centre of the mycelium and weaker cords, through the recycling and reallocation of the cytoplasm and biomass [83]. Individual, and a small number of co-located hyphae growing in the same confining areas appeared to have space searching as their primary objective, whereas the mycelium has much more complex biological objectives. Consequently, within the mycelium, various development pathways exist, e.g., the continuation of hyphal growth, the production of asexual structures, and progress into the sexual cycle, all of which can be expressed at the same time in different parts of the network [84].

Hardware. Many studies trying to elucidate the mechanisms responsible for mycelium extension—first performed as field work through the use of stable isotopes [85], and then increasingly using artificial constructs mimicking fungal environments both experimentally [77,86] and virtually [87,88,89]—are a rich source of information for understanding the mechanisms by which the fungal mycelium colonizes space. This ‘deputization’ of space searching is remarkable, as there is evidence that mycelia host complex interactions involving neuronal-like, electrical, and chemical communication [90] and that they also exhibit elements of memory of the direction of growth [91]. Fungi actively initiate movement within their network to transport both nutrients and other cellular contents depending on the environmental and local characteristics of their resource availability. It has been shown both at the macro- and microscales that fungi evolved an efficient way to communicate and translocate the required resources throughout the colony [92]. Large-diameter hyphae are used for long-distance nutrient translocation and signaling molecules, while fine side branches are specialized for nutrient uptake [29,85,93]. At the mesoscale [14], it was found that fungal network efficiency is modified when encountering moderate-to-rich resource patches and when growing in co-culture with bacteria, increasing the fractal dimension of the mycelium. As mentioned in the previous sections, the programs of both individual hyphae, like branching, as well as co-located hyphae, like cytoplasmic reallocation and negative autotropism, are all necessary for the ability of filamentous fungi to create interconnected networks; however, the mechanisms by which all these dynamic activities take place in a coordinated matter is still unknown. The physiological, molecular, and genetic mechanisms by which these activities take place were not spatially and temporally resolved at the mycelium level.

### 3.6. Multilayered Program Used by Filamentous Fungi for Space Navigation

#### 3.6.1. Information Processing in Spatial Navigation by Filamentous Fungi

When filamentous fungi are understood as information processing systems, similar to the model adapted for plant behaviour in a previous work [94], the external environmental conditions and their variations, i.e., chemical, physical, and biological, operate as inputs. These inputs are ‘processed’ by the organism, which responds accordingly, resulting in an observable output in the form of a morphological or physiological change, be that short or long, irreversible or reversible. This response or set of responses constitute the ‘external’ appearance of fungal behaviour, including the software, which is ‘hard-wired’ into the underlying biophysical, biochemical, biomolecular, and cellular mechanisms, the hardware. More specifically, the fungal software refers to the sets of biological and virtual procedures and instructions, i.e., natural algorithms, that result in its outputs, whereas the fungal hardware refers to the material mechanisms whose operation result in the observed behaviour.

#### 3.6.2. The Structure of the Fungal Program for Space Navigation

Our previous studies [16,17,18,36], which also corroborated with other reports [9,10,67], allowed for the construction of a general framework of the structure of the fungal program for space navigation in confining environments as follows.

(i) At the *single-hypha level*, it appears that three programs for space navigation, some with their own subroutines, are in play:

(i1) *Sensing* of a narrow passage by a hypha consists in the realignment of the hypha towards the narrow passage point in a space. This space searching program is directionality anisotropic, conditioned by *external*, extracellular parameters, and probably sensed by sensors placed on the hyphal membrane, followed by yet-to-be-determined transduction and actuation mechanisms. This program presents two subroutines:

- *Remote sensing*;
- *Contact-based sensing*.

(i2) *Directional memory*. This space searching program is anisotropic in nature, but driven by an internal mechanism consisting of the synergetic work of the Spitzenkörper and the microtubule cytoskeleton. It was shown [18] that the Spitzenkörper ‘points’ to the directionality the hypha had before its growth was deflected, and the microtubule cytoskeleton system ‘consolidates’ this memory.

(i3) *Branching*. This space navigation program consists of introducing alternative growing points to improve their ability to colonize ecosystems. It can be regulated by internal and external factors via two different subroutines.

- *Collision-induced*. Branching due to external sources, specifically collision-induced branching, is triggered when most or all physical avenues for growth are closed, e.g., corners, or when bluntly encountering obstacles. Because of the isotropic nature of turgor pressure, which appears to be its main driver, collision-induced branching presents a largely isotropic, opportunistic space search, especially in very small areas. Finally, collision-induced branching appears to have its own memory, manifesting as the gradual return to a lower branching frequency when encountering more open spaces.
- *Stochastic*. Fungi stochastically branch to enhance nutrient assimilation and interaction with their environment, partitioning the available space. This process is coordinated with the cell cycle through internal biochemical mechanisms.

At the *single-hypha level*, these three programs appear to operate independently and in parallel, with hyphal behaviour being the result of a ‘tug-of-war’ between them. From the perspective of space searching, sensing (especially its ‘remote’ variant) is the most beneficial due to the considerable shortening of the search time. Next, stochastic simulations demonstrated that directional memory results in faster and more comprehensive space searching [36], even when compared with artificial algorithms [95]. The opportunistic character of collision-induced branching should be placed at the bottom of the benefit scale of space searching for a *short time horizon*. While as a back-up, stochastic branching partitions space. However, collision-induced branching also operates as a backup mechanism when all the other subroutines fail and space searching needs to be rebooted. Consequently, collision-induced branching also consolidates the efficiency of space navigation considering a *long time horizon*. Notwithstanding these considerations regarding the efficiency of space searching, the ‘supremacy’ of one program or the other appears to be determined by the geometrical circumstances of the confining environment of the hypha. For instance, in dead-end corners, directional memory is turned off, and the sensing mechanisms (followed by transduction and actuation) are redundant. In this instance, collision-induced branching is the only useful, and thus operational, subroutine, working to emergency-reboot space searching.

(ii) At the level of *multiple*, *closely confined hyphae*, space navigation comprises two programs, that is, one implemented through external communication, and the other implemented internally.

(ii1) *Negative autotropism*. This program, which is prevalent when the respective hyphae are related via a considerably distant parent hypha, presents two variants, as dictated by the directionality vectors of their growth. If the hyphae have a near parallel direction of growth, and if the surrounding area allows it, they will redirect their growth away from each other, partitioning the available space. This program ensures the maximization of outward growth of the colony.

(ii2) *Cytoplasm reallocation*. This subroutine is prevalent when the hyphae are closely related, and therefore the distance between their apices is shorter than that for distantly related branches. A special discussion is required regarding cytoplasm reallocation. Cytoplasmic reallocation in filamentous fungi is still not entirely understood, specifically considering the intracellular mechanisms in which this optimization process takes place, thus requiring further research. Micro- [29], meso- [13,14,77,86,93], and macro-level [85] studies proved that fungi actively initiate movement within their network to transport both nutrients and other cellular contents depending on their local characteristics, such as resource availability and the presence of symbionts or competitors. In addition to the Brownian, diffusive mechanisms for solute transport in microorganisms, as well as biophysics [96,97] dominating tip growth in filamentous organisms [7], cytoplasm reallocation was shown to be a coordinated activity, dynamic within the different parts of the fungal network.

(ii3) *Anastomosis*. This program, consisting of the merging of two nearby hyphae, can start the subroutine initiating hyphal fusion to increase network robustness and share internal computational material for exploration.

At the level of *multiple, closely confined hyphae*, these programs are apparently determined by the level of ‘sibling’ relationship between the respective hyphae. From the perspective of energy consumption, the long distances needed for the reallocation of cytoplasm between distantly related hyphae makes this space searching subroutine costly. Conversely, closely related hyphae in tight, space-confining situations can perform a local space search by reallocating the cytoplasmic computational resource either through the directional change in cytoplasmic transportation or through the fusion of hyphae, without waiting for the resource to reach the leading hyphae from the farther regions of the colony. Unlike the programs at the single-hypha level, those for space navigation by multiple closely confined hyphae do not appear to operate in parallel or in coordination with each other. However, negative autotropism resembles *competition* between hyphae, whereas cytoplasm reallocation resembles *cooperation*.

(iii) At the *mycelial level,* the localized character of space searching is most evident, with the spatial distribution of fungal biomass being the result of a multitude of concatenated, but largely independent, local procedures for space searching, similar to scatter searching for optimization problems [98].

The above proposed framework for space navigation by filamentous fungi in confining spaces is not perfectly Cartesian, presenting many overlaps, both in terms of biological software and its hardware. Without being comprehensive, a few examples follow.

- Various levels of confinement can blur the distinction between *space partitioning*, prevalent in open spaces, and *space searching*, prevalent in tightly confining spaces. Indeed, while the navigation of mazes requires space searching subroutines, e.g., directional memory and collision-induced branching, the growth of fungi in larger, quasi-open chambers exhibit partially space partitioning characters, e.g., negative autotropism and stochastic branching.
- The space navigation programs for single and multiple co-located hyphae are ‘vertically’ connected, e.g., cytoplasm reallocation is often triggered by collision-induced branching, which itself is also triggered by the remote sensing of close by narrow passage points. Furthermore, while a ‘tug-of-war’ between the different programs manifests at the single-hypha level, it can also manifest ‘vertically’, e.g., when negative autotropism takes precedence over remote sensing and directional memory.
- There is a large, species-specific variability in the usage of space navigation subroutines, with some being turned “off”. For instance, while both *P. cinnabarinus* and *N. crassa* exhibit the collision-induced branching subroutine, *A. mellea* has it ‘turned off’ most of the time, as well as its directional memory.
- The framework proposed above does not explore, in detail, the impact of other biological software and their associated hardware, e.g., sporulation, which do not immediately contribute to space navigation by filamentous fungi.

**Figure 8:**
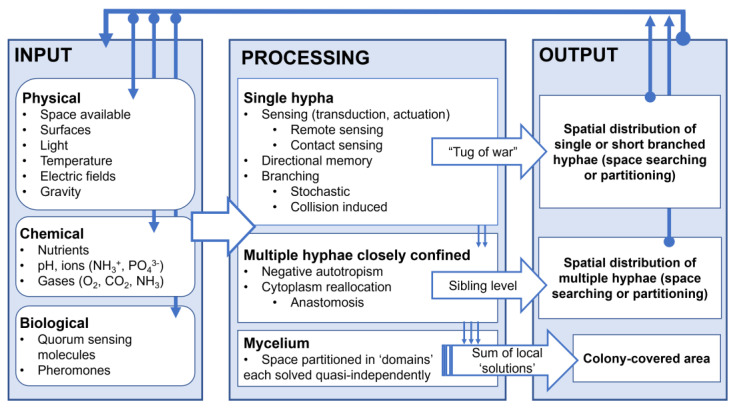
Information flow diagram for space navigation of filamentous fungi. *Information input:* Outside physical, chemical, and biological signals to be processed by single hypha or by multiple hyphae (collocated in the same confining space) or by the mycelium. *Information processing:* Multiple hyphae also benefit from the information input, both external and internal, from neighbouring hyphae. Information is sensed, transduced, and actuated in many instances by yet-to-be-defined pathways. *Information output*: First, for individual hypha, a response is morphological (easier to observe) or physiological change. This individual output may also feed into additional information input to multiple hyphae, which, in turn, produce outputs that impact ‘upstream’ the evolution of the mycelial network.

The interconnected structure of the information processing by filamentous fungi navigating space in confining environments is presented in Figure 8. The overall, qualitative assessment of the weight of the various space search subroutines at the levels of individual hyphae and multiple co-located hyphae is presented in Table 2.

The above description of the hierarchical structure of the space navigation subroutines suggest that each fungal level has a finite ‘intelligence capital’, which needs to be deployed optimally according to the extent and complexity of biological tasks ‘assigned’ at the respective level. Indeed, at the single-hypha level, this ‘intelligence capital’ seems to be entirely deployed for space searching, with the most important biological task resulting in a tug-of-war between the various subroutines. At the level of multiple closely confined hyphae, which need to triangulate their actions with each other, as well as perform additional tasks, a smaller proportion of the ‘intelligence capital’ can be made available for space searching, resulting in a simpler, on/off choice for a particular subroutine, i.e., negative autotropism or cytoplasm reallocation. Finally, at the mycelium level, the sheer number and complexity of biological tasks translates into essentially no part of the ‘intelligence capital’ being left available for space navigation.

### 3.7. Significance and Future Work

The major aim of the present work is to contribute to the growth of computer science and computer engineering via the reverse engineering of biological algorithms, in this instance, those used by filamentous fungi for space navigation. The final output of our work will be efficient algorithms and computer programs for optimization, in particular those related to the allocation of resources in space. Though it is far away, the final application of procedures based on conceptual paradigms developed significantly in the last decades such as soft computing [99] can be used to solve healthcare [100], biomedical [101], agricultural, and biological engineering problems [102]. However, while reverse engineering has been applied in synthetic biology [103], its application in mathematics and computer science is in its infancy.

Aside from this major aim focused on computer engineering deliverables, the present work may contribute to the understanding of how fungi colonize micro-confining environments, both with deleterious effects, e.g., the invasion of animal (including human) and plant tissues by pathogenic fungi, and with beneficial effects, e.g., symbiosis with plant roots. This research effort will require the extensive exploration of the modulation of fungal behaviour by environmental parameters, e.g., pH (with the possible use of pH-sensitive fluorescent dyes [104] for localized measurements at the microscale), temperature (with elaborate, better, non-invasive, local measurements [105]), pressure (possibly through the calibrated measurement of the deformation of PDMS structures [106]), and nutrient concentration and uptake (via the measurement of metabolites with oxygen-sensitive dyes [107]). Finally, the experimental and conceptual framework presented here can be applied to other motile microorganisms, e.g., bacteria, algae, and ciliates, thus offering enhanced opportunities for the reverse engineering of their respective biological algorithms.

## 4. Conclusions

The growth of several species of filamentous fungi in purposefully designed microfluidic networks revealed a general space navigation program, organized hierarchically at three levels: A single hypha, which consists of the most complex system of programs comprising sensing, directional memory, and branching; multiple closely confined hyphae, which use either negative autotropism or cytoplasm reallocation; and the mycelium, which does not possess a meta-program for space navigation, but delegates this task to sub-populations of hyphae. The hierarchical structure of the fungal space navigation program enables the efficient allocation of processing resources by aligning the increasing complexity of biological tasks—from a single hypha to the mycelium—with a decreasing need for navigational complexity at higher organizational levels. The efficiency of space navigation by filamentous fungi represents the synergy of the biological algorithms integrated into a hierarchical architecture of information processing, balancing complexity with specialization. These results suggest that expanding this line of research could lead to the discovery of mathematically efficient optimization procedures and the establishing of the field of informational biomimetics.

## Figures and Tables

**Table 1:** Growth characteristics of filamentous fungi in microfluidic structures.

Organism	*Pycnoporus cinnabarinus* [36]	*Armillaria mellea* [16]	*Neurospora crassa* [16,17]	*Neurospora crassa* ro-1 [17]
Phylum	Basidiomycota	Basidiomycota	Ascomycota	Ascomycota
Ecological niche	decomposer	plant pathogen	decomposer	dynein mutants, exhibit pleiotropic phenotypes
Leading hypha width (μm)	5.22 ± 0.74	3.86 ± 0.32	5.75 ± 0.50	5.30 ± 1.02
Apical extension velocity (μm/min)	1.63 ± 0.4	0.6 ± 0.2	0.8 ± 0.5	1.2 ± 0.7
Apical splitting	no	yes	yes	no
Branching distance CIB (μm)	81.4 ± 39.9	-	23.5 ± 15.0	17.7 ± 9.0
Lateral branching angle (°)	70.1 ± 14.6	54.6 ± 15.0	93.3 ± 15.4	89.4 ± 9.0
Angle before CIB (°)	<90	<70	<50	NA
Maze exit vs entrance rate	NA	0.56	0. 90	0.73

CIB: collision induced branching, NA: not available

**Table 2:** Various space navigation subroutines observed in studied filamentous fungi.

Fungal Level	Individual Hyphae					Co-Located Hyphae		
Software Species	Sensing Entries		Branching		Directional Memory	Cytoplasm Reallocation	Negative Autotropism	
	Contact	Remote	Collision-Induced	Stochastic			Side-by-Side	Head-on
*N. crassa*	very strong	strong	strong, backwards	strong	strong	weak	strong	not observed
*P. cinnabarinus*	strong	very strong	strong, at the tip	strong	strong	strong	very strong	very strong
*A. mellea*	strong	weak	none	very weak	weak	very weak	weak	none
*N. crassa ro-1*	strong	none	none	very strong	none	strong	none	none

## Data Availability

Movies, as well as more in depth protocols for the microfabrication procedures, can be found in the Supplementary Information.

## Supplementary Materials

The following supporting information can be downloaded at: https://www.mdpi.com/article/10.3390/biomimetics10050287/s1, SI1. Microfabrication protocols; Figure SI1. Example of the negotiation of concatenated symmetrical rectangular networks by the hyphae of *Aspergillus niger*; Figure SI2. Increasing branching distance of *N. crassa* in quasi-open space after traversing comb structures. SI3. Supplementary movie description of fungal species navigating microfluidic structures. Movie SM 01 *P. cinnabarinus* (RS, CIB), SM 02 *P. cinnabarinus* (RS, CS, CIB, DM), SM 03 *P. cinnabarinus* (CIB, CR, RS), SM 04 *P. cinnabarinus* (CS, DM, CIB, RS), SM 05 *A. mellea* (CS, CIB off), SM 06 *P. cinnabarinus* (CIB, RS, CR, NA, CS), SM 07 *N. crassa* (RS, CIB), SM 08 *A. mellea* (CIB off, NA off), SM 09 *P. cinnabarinus* (NA, CS, DM, RS), SM 10 *A. mellea* (RS, CS, NA off) and SM 11 *P. cinnabarinus* (CR, NA) [Remote Sensing (RS), Directional Memory (DM,) Contact Sensing (CS), Collision-Induced Branching (CIB), Negative Autotropism (NA), and Cytoplasm Reallocation (CR)].
