# Supplementary material for: Hierarchical Structure of the Program Used by Filamentous Fungi to Navigate in Confining Microenvironments
Source: Biomimetics (Basel). 2025 May 2;10(5):287. doi: 10.3390/biomimetics10050287 (PMC12109565; doi:10.3390/biomimetics10050287)
Supplement: Supplementary file 1 [file biomimetics-10-00287-s001.zip › biomimetics-3573939-supplementary.pdf]

Hierarchical structure of the program used by filamentous fungi to navigate in confining microenvironments

Gala Montiel-Rubies, Marie Held, Kristi L. Hanson, Dan V. Nicolau Jr., Radu C. Mocanasu, Falco C.M.J.M. van Delft, and Dan V. Nicolau

SUPPLEMENTARY INFORMATION

SI 1. Microfabrication protocols

The fabrication process contains the following steps:

1. Fabrication of a monolithic casting master in silicon

a. A 4-inch silicon wafer was covered with a 520 nm thick SiO_2 layer by PECVD at 300°C.
b. A hexamethyldisilazane (HMDS) prime layer was spin-coated at 2000 rpm and baked on a hotplate at 200°C for 2 mins.
c. A 200 nm thick NEB22 (Sumitomo) resist layer was spin-coated at 2000 rpm and pre-baked on a hotplate in an oven at 110°C for 2 min.
d. Electron beam exposure on a VISTEC-5200 system at 100 kV, with a 17 $\mu C/cm^2$ dose. For coarse patterns, 100 nm beam step size, 150 nm spot size (52 nm defocus), 148 nA beam current, and for fine patterns, 25 nm beam step size, 40 nm spot size (8 nm defocus), 7 nA beam current.
e. Post-exposure, bake on a hotplate in an oven at 105 °C for 2 min.
f. Vertical development for 30 s in MF322, rinsed twice for 15 s in fresh MF322:H_2O=1:10, rinsed twice for 15 s in fresh H_2O.
g. Spin-dried at 2000 rpm.
h. SiO_2 etched in a pre-conditioned AMS 100 I-speeder Bosch system, using 20 sccm C4F8, 100 sccm He and 10 sccm CH_4 flow, with RF1=2500 W, RF2=300 W, 100% pumping, SH position source: 120 mm, chuck temperature 0°C, and etch time 3 min 10 s.
i. 2 µm deep Si-etch in ICP Chlorine system, with 9 sccm Cl_2, 1 sccm O_2, 7 µbar, RF power 100 W, chuck temperature 65°C, and etch time 15 min. 18 s; OR
j. 5µm deep Si-etch in AMS cryo-etcher system with chuck temperature -120°C, RF1=1100 W, LF generator pulsed (40 W 10 ms/W 90ms),100% pumping, using:
k. 1 cycle of [26 sccm O_2 and 200 sccm SF6 flow] Power 13 s ON + 5min. OFF (cooldown)
l. 6 cycles of [28 sccm O_2 and 200sccm SF6 flow] Power 15 s ON + 5min. OFF (cooldown)
m. The NEB22 residue was removed in a Reactive Ion Etching system with 20 sccm O_2 at 30 µbar, with 50W RF power 1 min 30s etch time.
n. The SiO_2 residue was removed in buffered HF solution (1:7) for 2 min. 30 s, followed by demi-water rinse for 5 min, and spin-dried at 3000 rpm.
o. SEM inspection

2. The casting of a PDMS replica from the monolithic Si master, dicing and release

a. A small amount (0.5-1ml) of chloro-trimethyl-silane in a small conical flask was placed together with the silicon wafer in a vacuum chamber. After releasing the vacuum, the vessel was opened to let silanization occur for approximately 2 hours.
b. Sylgard 184 kit-polydimethylsiloxane (PDMS) was mixed in a 10:1 ratio (monomer: crosslinker) and stirred ~ 3min.
c. The PDMS mixture was poured over the master structure (all in a Petri dish) and placed in a vacuum chamber to remove air bubbles (~2 hr.)
d. The Petri dish (with master covered in PDMS) was placed in the oven overnight at 65°C or kept under room temperature for curing 1-2 days.

e. The chip was cut around the structure and slowly peeled off from the master (the master is then usually covered with a new PDMS mixture to keep it free from contamination (followed by steps b-d).

3. Sealing the replica with an oxygen-permeable PDMS cover

a. The PDMS casts (structure face up) and coverslips were put in a plasma cleaner (Harrick Plasma, PDC-32G), pumped down and exposed for 30 s - 45 s.
b. After plasma treatment, immediately, the casts were turned upside down (structure face down) over the exposed coverslips for sealing.
c. After the chip was sealed, the whole unit was placed in a Petri dish with distilled water or malt extract broth in a vacuum chamber for at least 5-10 min to ensure the medium wetted the structures.
d. The chips were stored at 4 °C for future use.

SI 2. Example of growth patterns of *Aspergillus n*iger in microfluidic structures.

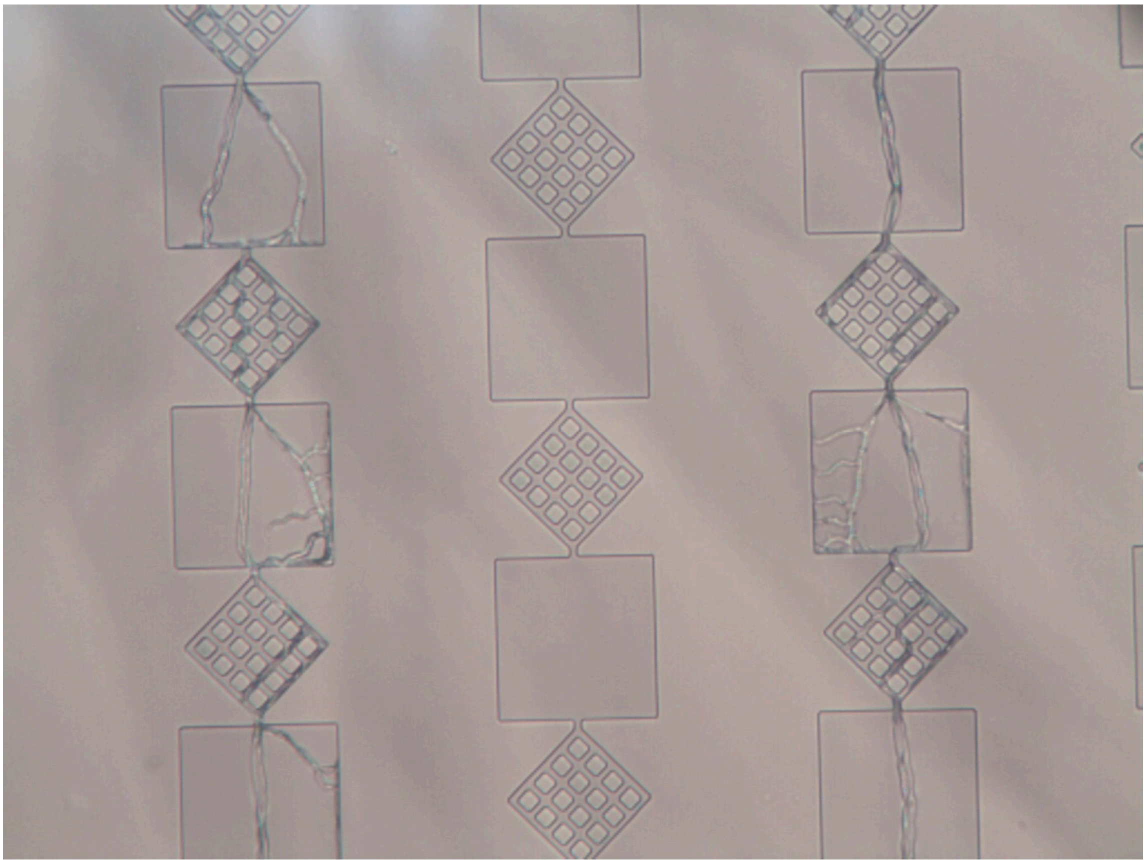

Figure SI 1. Example of the negotiation of concatenated symmetrical rectangular networks by hyphae of *Aspergillus niger*. All space searching algorithms used by other species studied here can be clearly observed, (i) for individual hyphae (*remote sensing*: left column/middle chamber, right column/top chamber; *contact sensing*: left column/top chamber; *collision-induced branching*: left column/middle chamber; *directional memory*: left column/bottom chamber, right column/top and bottom chamber) and (ii) for hyphae confined together (*cytoplasm reallocation*: left column/top and middle chamber; and *negative autotropism*: left column/all chambers, right column/middle chamber).

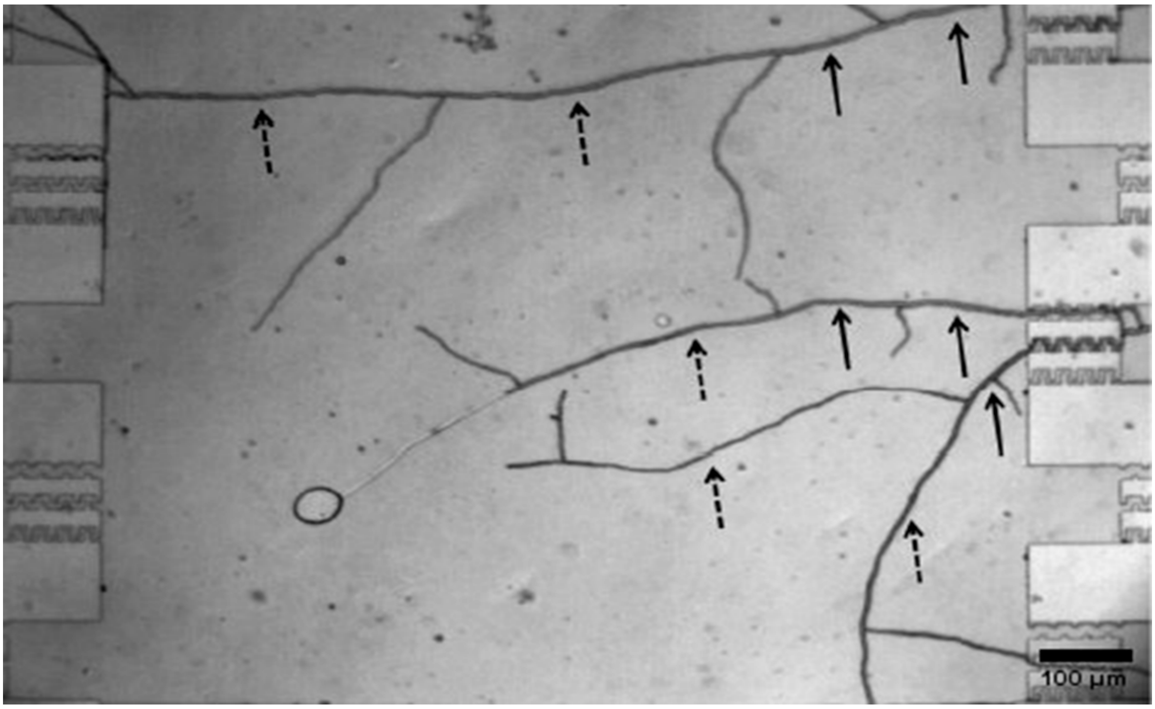

Figure SI 2. Increasing branching distance of *N. crassa* in quasi open space after traversing comb structures. These gradually increasing branching distances indicate that the hyphae undergo a recovery phase from the structure.

SUPPLEMENTARY INFORMATION: SUPPLEMENTARY MOVIES

SI 3. Supplementary movie description of fungal species navigating microfluidic structures.

Movie	Species, searching subroutines	Description
Movie SM 01	*P. cinnabarinus* RS, CIB	All three exploring hyphae (1,2 and 3) remotely sense (RS) the narrow entry to the complex maze structure. Inside the structure hypha 1 gives rise to daughter hypha (4) due to collision induced branching (CIB).
Movie SM 02	*P. cinnabarinus* RS, CS, CIB, DM	Single hypha (1) explores microfluidic structures. Through remote sensing (RS) it precisely enters the second symmetrical rectangular network. When encountering a corner, it is bounded for a few minutes, giving away to branching due to collision induced branching (CIB). When entering the third maze it uses contact sensing (CS) to readjust its entrance. This time the hypha explores the checkered obstacles and initializes its extension with a degree of directional memory (DM) that gets lost briefly after. When exiting the fourth time, it is also stuck in a corner which induces collision induced branching (CIB), hypha 2 remotely senses (RS) the entry of the narrow passage to start exploring. This daughter branch (2) maintains its directional memory (DM) through the diamond structure while hypha 1 reallocates its cytoplasm (CR) to deliver cytoplasmic contents to the leading hypha.
Movie SM 03	*P. cinnabarinus* CIB, CR, RS	The leading hypha (1) collides with the wall and branches (2) and (3) due to collision induced branching (CIB). The small daughter hypha (2) ceases growing, and hypha (3) enters the microfluidic maze. A few minutes after, there is a process of cytoplasm reallocation (CR) to promote the exploration of new space. New branches introduce to the system (4-8). Hypha 4 redirects its growth through remote sensing (RS) to adjust its introduction to the narrow entry.
Movie SM 04	*P. cinnabarinus* CS, DM, CIB, RS	Leading hypha (1) contact senses (CS) the narrow entry to the complex maze. Inside the structure it starts with a distinguished pattern when traversing the obstacles, showing directional memory (DM). This is lost when it is blocked by a corner. Two daughter branches (2 and 3) appear due to collision induced branching (CIB). Branch 2 gives rise to several daughter branches due to this same mechanism. New exploratory hyphae (3 and 5) enter the structure and repeat the contact sensing (CS) behaviour shown by hypha 1. However, hypha 3 enters the narrow entry through remote sensing (RS) when it branches to hypha (6) precisely in the entrance. Meanwhile, in the maze new branches keep emerging due to collision induced branching (CIB) as a prevailing strategy to colonize more space.
Movie SM 05	*A. mellea* CS, CIB off	*A. mellea* hypha (1) enters channel through contact sensing (CS). It continues without branching through the long straight path, even when encountering a corner.
Movie SM 06	*P. cinnabarinus* CIB, RS, CR, NA, CS	There is collision induced branching (CIB) of leading hypha when it encounters a corner and produces two daughter hyphae (2 and 3) that do not extend further. Branch 4 remotely senses (RS) the narrow entry to enter the symmetrical rectangular network. As it extends hyphae 2 and 3 reallocate their cytoplasm (CR) to promote growth of hypha 4 that branches (5) in a narrow opening. Branch 4 and branch 5 separate from each other due to negative autotropism (NA) after exiting the maze. Both hyphae collide with the corners of the structure and branch due to collision induced branching (CIB), which produces branches 7 and 8. Daughter hypha 6 contact senses (CS) the opening to the next structure.
Movie SM 07	*N. crassa,* RS, CIB	Three hyphae exit the first complex maze towards open space. Hypha 1 remote senses (RS) the narrow entry branching precisely to enter the second

		maze. Once inside hypha 4 branches through collision induced branching (CIB) to increase the number of exploration paths giving way to branches 5, 6 and 7.
Movie SM 8	*A. mellea* CIB off, NA off	A single exploring hypha gets to the exit channel of the complex maze without the aid of branching.
Movie SM 9	*P. cinnabarinus* NA, CS, DM, RS	Two hyphae enter the open space and maintain an avoiding behaviour due to negative autotropism (NA). Both hyphae enter the symmetrical rectangular network based on contact sensing (CS), however hypha 1 traverses through the obstacles presenting directional memory (DM), while hypha 2 prefers to nest the margin walls. After exiting the maze, the hyphae present again negative autotropism (NA). This time hypha 1 enters the narrow entry to the next structure through remote sensing (RS), while hypha 2 gets stuck in the corner without branching, pushing hypha 1.
Movie SM 10	*A. mellea* RS, CS, NA off	Two exploring hyphae enter the symmetrical rectangular network. Hypha 1 enters through the narrow entry by remote sensing (RS) while hypha 2 finds its way through contact sensing (CS). Both hyphae traverse the grid using the external walls without branching, even when encountering corners. When exiting the maze towards open space they avoid each other due to negative autotropism (NA). Hypha 2 does not sense the narrow entry (arrow) and eventually hyphae 1 and 2 reencounter (circles), disregarding an avoidance behaviour or negative autotropism (NA).
Movie SM 11	*P. cinnabarinus* CR, NA	Hypha 1 and hypha 2 enter the symmetrical rectangular network through opposite directions. When getting close to each other (circle) hypha 2 ceases extension while hypha 1 starts branching in different places. When hyphae 1 and 3 continue their extension, hypha 2 retreats (NA) and initiates a process of cytoplasm reallocation (CR).

Note: Space searching subroutines were denominated as: Remote Sensing (RS), Directional Memory (DM,) Contact Sensing (CS), Collision-Induced Branching (CIB), Negative Autotropism (NA), and Cytoplasm Reallocation (CR).
